# Taking note: A qualitative study of implementing a scribing practice in team-based primary care clinics

**DOI:** 10.1186/s12913-019-4355-z

**Published:** 2019-08-14

**Authors:** Jennifer M. Van Tiem, Kenda R. Stewart Steffensmeier, Bonnie J. Wakefield, Greg L. Stewart, Nancy A. Zemblidge, Melissa J. A. Steffen, Jane Moeckli

**Affiliations:** 1 VISN 23 Patient Aligned Care Team Demonstration Lab, Iowa City VA Health Care System, 601 Hwy 6 West, Building 42, Iowa City, Iowa, 52246 USA; 2 CADRE, the Center for Comprehensive Access and Delivery Research and Evaluation, Iowa City VA Health Care System, 601 Hwy 6 West, Building 42, Iowa City, Iowa, 52246 USA; 3 Tippie College of Business, University of Iowa, 21 E Market St, Iowa City, Iowa, 52242 USA; 4 Sinclair School of Nursing, University of Missouri, S313 School of Nursing, University of Missouri, Columbia, MO 65211 USA

**Keywords:** Medical scribes, Normalization process theory, Primary care, Qualitative methods, Team-based care

## Abstract

**Background:**

Though much is known about the benefits attributed to medical scribes documenting patient visits (e.g., reducing documentation time for the provider, increasing patient-care time, expanding the roles of licensed and non-licensed personnel), little attention has been paid to how care workers enact scribing as a part of their existing practice. The purpose of this study was to perform an ethnographic process evaluation of an innovative medical scribing practice with primary care teams in Veterans Health Administration (VHA) clinics across the United States. The aim of our study was to understand barriers and facilitators to implementing a scribing practice in primary care.

**Methods:**

At three to six months after medical scribing was introduced, we used semi-structured interviews and direct observations during site visits to five sites to describe the intervention, understand if the intervention was implemented as planned, and to record the experience of the teams who implemented the intervention. This manuscript only reports on semi-structured interview data collected from providers and scribes. Initial matrix analysis based on categories outlined in the evaluation plan informed subsequent deductive coding using the social-shaping theory Normalization Process Theory.

**Results:**

Through illustrating the slow accumulation of interactions and knowledge that fostered cautious momentum of teams working to normalize scribing practice in VHA primary care clinics, we show how the practice had 1) an organizing effect, as it centered a shared goal (the creation of the note) between the provider, scribe, and patient, and 2) a generative effect, as it facilitated care workers developing relationships that were both interpersonally and inter-professionally valuable. Based on our findings, we suggest that a scribing practice emphasizes the complementarity of existing professional roles, which thus leverage the interactional possibilities already present in the primary care team. Scribing, as a skill, forged moments of interprofessional fit. Scribing, in practice, created opportunities for interpersonal connection.

**Conclusions:**

Our research suggests that individuals will notice different benefits to scribing based on their professional expectations and organizational roles related to documenting patient visits.

**Electronic supplementary material:**

The online version of this article (10.1186/s12913-019-4355-z) contains supplementary material, which is available to authorized users.

## Background

Medical scribing and its burgeoning infrastructure in the United States – scribing companies, training and certifying organizations, advocacy groups – comprise an industry with a predicted 100,000 practicing scribes by 2020 [1]. The aim of medical scribing is to improve healthcare delivery and outcomes by adding a care worker to the healthcare team to document patient-provider encounters in the electronic medical record under clinician supervision [1]. Scribes could be personnel who function solely as scribes (e.g., “documentation specialists”), but they could also be team members, like medical assistants, who take on scribing as an extension of their existing duties [2–4]. There are nascent standards for scribe training championed by the American College of Medical Scribe Specialists; however, it is still common that individuals who function as scribes are not certified as such, and have instead achieved variable levels of education, from a high school degree to a licensure in vocational/practical nursing [5]. Despite variable training, “scribes are personnel specifically hired to chart patient-clinician encounters in real time, from the beginning of the encounter to the end” [6].

Early literature examines the impact of medical scribes in emergency departments and specialty clinics [7–10], while more recent studies follow the progression of scribes in primary care settings [11–17]. Beneficial outcomes attributed to scribing include: reducing documentation time, improving note quality, improving workload capture [13, 17–21], increasing patient and provider satisfaction, and improving provider work life by reducing burnout [7, 9–12, 14–17, 22, 23]. Though much is known about benefits attributed to medical scribes, little attention has been paid to the work clinicians do to implement and maintain a scribing practice. Because the recent increase in scribing is driven largely by attempts to ease burnout associated with the use of electronic health records [4], more ethnographic knowledge is needed about what scribing entails in practice. The aim of this manuscript is to describe medical scribing and the work involved to implement a scribing practice by presenting findings from an ethnographic process evaluation that was designed to identify barriers and facilitators to introducing scribing into primary care teams in Veterans Health Administration (VHA) clinics.

Over the past decade in the United States, the VHA has striven to transform the delivery of primary care services through the implementation of a patient-centered medical home initiative [24–30]. Called Patient-Aligned Care Teams (PACTs) in VHA, PACTs are comprised of one or more small teams that bring a single provider, Registered Nurse (RN), Licensed Practical Nurse (LPN), and clerk together to coordinate the care of a panel of approximately 1200 Veteran patients [31, 32]. PACT is advocated as an approach to team-based care that expands the roles of non-provider support staff, and in so doing helps overcome negative trends such as provider staffing shortages and provider burnout [3, 33–37]. To enhance both the provider and patient experience of primary care, VHA Primary Care Services engaged in a proof of concept scribe program intended to improve patient-provider communication and reduce provider documentation time. We conducted an ethnographic process evaluation [38, 39] of this program, called the Health Advocate Demonstration Project, as it was implemented at different clinics over the course of 2 years.

As part of the Health Advocate Demonstration Project, teams hired licensed and non-licensed personnel to fill the scribe role. Scribes were asked to document a patient’s history of present illness, review of systems, physical exam, lab results, treatment plans, and discharge information. Scribes were also encouraged to track laboratory and imaging tests, communicate lab findings to the provider, and complete medical documentation as instructed by the provider. In these ways, scribing in VHA primary care clinics was typical of scribing more broadly. The aim of the ethnographic process evaluation of the Health Advocate Demonstration Project was to document the barriers and facilitators to implementing a scribing practice in primary care clinics, as well as to develop an understanding of team members’ perceptions of the practice, the impacts of the practice on team function and workflow, patient and provider satisfaction, and more broadly the quality of patient-provider communication. Our findings address the need to better understand the role and significance of scribes on health care practice [6] and build on more recently published qualitative descriptions of primary care workers’ perspectives on scribing [15, 16].

Using ethnographic data collection methods and Normalization Process Theory as our analytic framework, this manuscript reports on the slow accumulation of interactions and knowledge that fostered cautious momentum of primary care teams working to normalize scribing practice. Normalization Process Theory (NPT) is a model of implementation that encourages paying attention to the mundane social processes that change to support the implementation of an innovation. It was originally developed to understand the implementation of “complex healthcare interventions” and has since been employed in over 100 studies to understand the feasibility of healthcare interventions [40, 41]. We used a construct of NPT, “collective action,” to follow how care workers in VHA primary care clinics mobilized the necessary skills and resources to implement a scribing practice [42–47]. By illustrating these incremental changes involved in introducing a scribe in VHA primary care clinics, we show how this innovative practice had 1) an organizing effect, as it centered a shared goal (the creation of the note) between the provider, scribe, and patient, and 2) a generative effect, as it facilitated relationships among care workers that were both interpersonally and inter-professionally valuable.

## Methods

### Aim

The aim of this manuscript is to describe medical scribing and the work involved to implement a scribing practice by presenting some of the findings from an ethnographic process evaluation of the Health Advocate Demonstration Project, which introduced scribes into primary care teams in VHA clinics. Our ethnographic process evaluation was designed to develop an understanding of team members’ perceptions of scribing, the impacts of scribing on team function and workflow, patient and provider satisfaction, and more broadly the quality of patient-provider communication. The supporting research was performed in cooperation with VHA Patient Care Services and received a Quality Improvement designation and determination of non-Human Subjects Research from the University of Iowa Institutional Review Board (IRB #201609852).

### Design

Our evaluation team, composed of experts in anthropology, nursing, and public health, conducted the process evaluation of the Health Advocate Demonstration Project using a rapid ethnographic approach [38, 39, 48], collecting data at different time points before and after launch (September 2016–June 2018). We participated in and took notes during bi-weekly calls led by Patient Care Services for sites participating in the Health Advocate Demonstration Project. During these calls, representatives from Patient Care Services and site-level facilitators asked and answered questions about the implementation of the project as sites encountered barriers to their progress. We conducted baseline telephone focus groups with site-level facilitators at all 13 sites that planned to implement the scribing intervention. We recorded how they were preparing to implement the intervention, including what resources they were marshalling. Finally, because site-level facilitators may or may not have been providers or scribes implementing the intervention, we conducted five site visits during which we used semi-structured interviews and direct observations to describe the intervention, understand if the intervention was implemented as planned, and to record the experience of the teams who implemented the intervention. The timeline of our ethnographic process evaluation (January 2017–April 2018) coincided with the implementation of the Health Advocate Demonstration Project (September 2016–August 2018). This manuscript only reports on semi-structured interview data collected from providers and scribes who implemented a scribing practice during the site visit phase of our evaluation.

### Setting & characteristics of participants

Of the 13 sites that planned to implement the Health Advocate Demonstration Project, only six launched and sustained a scribing practice during the site visit phase of our evaluation (August 2017–March 2018). Most sites accepted into the Health Advocate Demonstration Project were unable to start-up a scribing practice during the term of the evaluation (September 2016–June 2018) because of organizational barriers, such as leadership buy-in, turnover, provider buy-in, hiring, and the availability of equipment and space. Due to timing and budgetary constraints, we conducted a site visit at five of the six sites that launched and sustained a scribing practice. Because we were focused on provider-staff communication, we report here only on data where we were able to talk with members from both groups. Thus, the data reported here are only from sites where we performed site visits. When we were on site, no one declined to speak with us. We visited three VHA Medical Centers and two VHA Outpatient Clinics that housed 8 individual PACT teams. Table 1 organizes details about these 8 teams.

**Table 1:** Characteristics of Visited Teams

Team	Medical Center or Outpatient Clinic	Location	Team Composition^a^	Provider^a^	Scribe^a^	Launch Date	Site Visit
1	Medical Center	West	MD, RNCM, LPN1, LPN2	MD	LPN1	Dec 2016	Aug 2017
2	Outpatient Clinic	Midwest	DO, RNCM, LPN, MSA	DO	LPN	Apr 2017	Dec 2017
3a	Medical Center	East	NP, RN, LPN	NP	LPN	May 2017	Sept 2017
3b			MD1, MD2, MD3, LPN1, LPN2, LPN3, RN, RN2, MSA	MD1, MD2	LPN1	Jul 2017	Sept 2017
3c			MD, LPN1, LPN2, RN	MD	LPN1	Jul 2017	Sept 2017
4	Medical Center	West	NP, RNCM, LPN, CNA, MSA	NP	CNA	Sept 2017	Mar 2018
5a	Outpatient Clinic	Midwest	DO, RNCM, LPN1, LPN2, MSA	DO	LPN1, LPN2	Nov 2017	Mar 2018
5b			MD, RNCM, LPN, MSA	MD	LPN	Nov 2017	Mar 2018

^a^Please see List of Abbreviations

### Data collection

Data include interviews with providers and scribes from the five sites where we conducted site visits. An outline of our semi-structured interview guide used with providers and scribes is included in Additional file 1. Semi-structured interviews were conducted by JVT, KSS, MS, and BW. We audio-recorded each interview on encrypted voice recorders and JVT, KSS, and NAZ transcribed the interviews. All interview materials were uploaded into a structured codebook in Microsoft Word (Additional file 2) in preparation for a matrix analysis [49]. These forms were populated with interview data and then uploaded to MAXQDA v18 [50] for additional deductive analysis.

### Data analysis

Initial matrix analysis [49] of all transcripts was carried out by JVT and KSS based on categories outlined in the evaluation plan (e.g. site, role, facilitators, barriers, team function, impact of the model). Interview data from each PACT team were transcribed directly into a structured codebook (Additional file 2) and saved as Microsoft Word files. Decisions about coding were made twice: first when initially sorting the data into categories while transcribing the interview, and then second, when writing up a site visit report to give back to the site. JVT and KSS transcribed and coded separately initially, then reviewed the documents for each team together, and finally wrote the site visit reports together. Through completion of five site visit reports, JVT and KSS noticed patterns related to how the providers and scribes built their scribing practice together. Using the “Structured Text” function of MAXQDA v18, JVT then uploaded each of the forms populated with interview data into MAXQDA v18 for additional analysis using Normalization Process Theory (NPT).

Each of the categories in the structured codebook became a code in MAXQDA. In consultation with a senior social scientist familiar with NPT (JM), JVT deductively re-coded interview data with scribes and providers using the four elements that comprise “collective action” within the framework of NPT [46, 47]: contextual integration; skill-set workability; interactional workability; and relational integration. Table 2 presents definitions for each element, as outlined in Holtrop et al. (2016) [47]. Segments sub-coded as one of the four elements were then sorted into smaller groups, as sub-categories of elements of collective action. Sub-categories, also outlined in Table 2, were used to re-code each segment to generate a grounded understanding of collective action in the context of the data from the five site visits with VHA primary care providers and scribes. We expected that the construct of collective action, as an analytical lens, would help us develop specific descriptions of the skills and resources required to successfully implement a scribing practice.

**Table 2:** Elements of Collective Action, their Theoretical Definition, and their Grounded Usage

Elements of Collective Action	Theoretical Definition	Grounded Sub-Categories
Contextual Integration	“The fit between the new intervention and the overall organizational context” [47]	• Addressing regulatory concerns about documentation requirements • Making a template note • Using the electronic medical record
Skill-Set Workability	“The fit between the new intervention and existing skill sets” [47]	• Sharing the note • Matching physician and scribe roles • Deciding when to scribe
Interactional Workability	“The impact a new intervention has on interactions, particularly the interactions between health professionals and patients” [47]	• Learning and managing differences and preferences between physicians and scribes • Noticing changes in interactions with patients
Relational Integration	“The impact of the new intervention on relations between different groups of professionals; includes issues of power and trust” [47]	• Maintaining interprofessional connections between the physician and scribe • Encouraging interpersonal connections between the scribe and patient • Trusting those connections to support primary care practice

## Results

The following results sections reports only on the analysis of semi-structured interviews (*n* = 18) with provider and scribe teams. Table 2 summarizes the inductively defined sub-categories associated with each element of collective action. Broadly, sub-categories associated with contextual and relational integration addressed work done at the system level to institutionalize the program, as well as work between staff and patients to build and maintain work and care relationships. Sub-categories associated with skill-set and interactional workability addressed work by staff at the individual level defining, delegating, and learning roles, as well as navigating interactions.

### Scribing had an organizing effect

The work of creating the template for documenting patient encounters in the electronic medical record helped providers and scribes learn and negotiate how to complete the note with each other. Primary care providers and scribes were acutely aware that the note was a legal document and needed to adhere to The Joint Commission guidelines. Clinical staff also acknowledged documentation standards of VHA, including attending to national VHA priority reminders, meeting peer review requirements, and formatting the note for coding and billing. Some primary care providers and scribes worked with information technology specialists to develop note templates that addressed documentation requirements while also meeting the provider’s preferences for the note, including how to organize sections of the note and what information to include in each section.

Creating the note template acculturated the scribe to note-taking through an iterative process that involved reviewing the provider’s prior notes and working with the provider to divide responsibility for generating the distinct parts of new notes (e.g., Review of Systems, Physical Exam, History of Present Illness, Assessment and Plan). Scribes and providers had to negotiate what the scribe recorded (e.g., history, assessment) and how that was recorded (e.g., summary, verbatim, shorthand) to create an integrated note. When asked how she knew that the scribe was ready to take more responsibility in creating the note, one provider reflected,

“Because she was hitting the highlights she was learning what I was looking for, and what I thought was really important to include in the note, and what to emphasize and how to organize it…” (NP, Site 3a)

Sharing the note looked different for each provider-scribe team. Providers and scribes had to work to figure out how to share a voice with each other, as well as the patient, in the note. Some providers were concerned about the note continuing to sound like it was written in their voice. From the provider’s point of view, one provider noted how, “You don’t want to make [the notes] too wordy and boggy that people aren’t going to read them, but I need them to relay my point” (DO Site 2). Scribes, on the other hand, were learning to balance the provider’s voice with the patient’s words. A scribe reflected,

“It’s hard to figure out what is subjective versus what is actual, like [what the provider’s] trying to put in there. So, if [the patient is] saying you know, ‘I have right hip pain because I broke my hip,’ … it’s just, sometimes difficult to get it in there in their words.” (CNA Site 4)

In managing these uncertainties, teams often chose to pilot their scribing practice with acute appointments, “…just because it’s just more direct and it’s more simple to learn…the importance of what we need to get into the note” (LPN Site 5b). Teams would then slowly incorporate annual visits and new patient visits into their scribing practice. In deliberately deciding how, when, and where the scribe would enter the process of making the note, providers were acutely aware of how they benefitted from a more fully integrated scribing practice. One provider noted that in moments when scribing went well, she was able to, “spend more face-to-face time with the patient, getting to know them,” and have “more meaningful time writing the assessment and plan,” with the scribe already “having that supporting document be *done*” (MD Site 5b, participant’s emphasis).

### Scribing had a generative effect

With an eye to institutional and organizational requirements for the note (e.g., a legal document, an archival record, a catalogue of information, etc.), providers and scribes not only delegated note-taking tasks based on roles and skills, but they also learned and managed each other’s conversational differences, writing styles, and organizational habits. As they continued to make deliberate efforts to understand each other and generate a coherent note, they noticed changes in their interactions with each other. One provider-scribe team reflected how,

*Scribe:*

“We definitely talk different. You know, he wants it in *his* words and that’s where sometimes it is hard because I’m not a provider, I don’t talk like a provider, but I’m learning how to type like a provider. [Interviewer: What does it mean to talk like a provider?] Medical terms, umm, total knee replacement, that’s not a medical term, you know… So, what he’ll do, is he’ll say to the patient ‘total knee replacement,’ but he wants in his *note*, the medical term. So, he’ll do it in layman’s terms for the patient because, *I* would need that, but he wants his note to sound professional.” (CNA Site 4, participant’s emphasis)

*Provider:*

“So, the scribe herself is a sharp person but, she’s not well educated…and one of the things I’ve done is just have her put words in the review of systems…put ‘back pain,’ you know, do that, and then when I go back down I can go into the review and just put ‘He had back pain with blah blah blah…’ But she does have a standard…thing on her template… and I had to learn how to like talk to her too, but addressing the patient, ‘Oh your lungs are clear. Your heart sounds…You have a murmur.’ And that seems to help her.” (NP Site 4)

*Scribe:*

“Yeah, so you know I’ve learned to google a lot [for medical terms] while I’m in there…[and] I still, before every patient I go through and look at what [the provider has] done before. And I copy and paste, not like his note from last year, but I put stuff that I know he’s gonna ask…so when I’m on my note, I can see what he’s looking at.” (CNA Site 4)

Providers and scribes were called to recognize and accept the differences in each other’s knowledge base and educational background that might impact how they both needed to use the note differently in their own work in the future. The scribe, using a template and copying information forward, wanted to be able to follow the conversation between the provider and the patient during the appointment. The provider, wanting the note to sound professional, perhaps had an eye toward peer review.

Sharing the note and sharing the work of creating the note unfolded into interprofessional connections between the provider and scribe and interpersonal connections between the scribe and patient. The interprofessional connections between the provider and scribe built into mutuality. Mutuality between the provider and scribe opened the door to a kind of leveling between the provider and patient. In the context of an appointment, one provider-scribe team described how,

*Scribe:*

“Like [the provider will] say, ‘Do you have any questions while I’m going through stuff with the patient, please stop me and ask me’ you know, as part of my scribing. So, I feel very comfortable, like if I didn’t hear something, or I have a question about something, I feel comfortable stopping and asking her and she’s more than willing to answer it for me. In turn, sometimes she’ll say to me, ‘Is there anything that I forgot to ask them that you can think of, you know as part of their history that I might have forgotten.’ There’s been times when I’ll say, ‘Well we didn’t ask this question,’ and she’s like, ‘Oh yeah! Thanks for reminding me.’” (LPN Site 2)

*Provider:*

“I think in the long run, as we continue to work together, it’s almost intuitive, what I’m thinking, and the next question I’m going to ask. And I’m gracious when she says, ‘Oh remember, we talked about this last time, are you going to ask him about this, or ask her about this?’” (DO Site 2)

Working collaboratively to figure out how they would scribe allowed the provider to re-engage the patient. Though pitched as a practical solution to the problem of snowballing documentation responsibilities, the effort to embed scribing in PACT teams highlighted the promise of team-based care to be patient-centered by creating opportunities for meaningful work for providers and scribes, and potentially more engaged encounters with patients. In answering the question, “What did you hope to gain or achieve by participating in the pilot,” one provider responded,

“Being able to be more present with my patients. I love the electronic medical record in many ways, [but] I don’t like that my view is here [at the computer] and not with patients, because just that little bit of eye contact makes a huge difference. Even if I might be intently looking for something, patients interpret that as ‘You’d rather look at a screen.’ My goal was to make patients feel ‘I’m here for you, I’m present for you, I’m choosing to come here.’… I prefer to have more face to face time because I find that patients talk to me more. I’ll get the ‘Oh by the ways,’ [like] ‘I’ve been having chest pain when I walk my dog.’ I’ve found that it’s helped a lot because now I can be eye-to-eye with patients again.” (DO Site 2)

## Discussion

Though current literature suggests that introducing scribing does influence outcomes that can be measured and described quantitatively (e.g., provider burnout, patient satisfaction), our qualitative methods afforded us the opportunity to pay attention to the slow accumulation of interactions (i.e., how to write the note) and knowledge (i.e., what goes in the note), and thus track how teams negotiated as they worked to enact a scribing practice. Scribing had an organizing effect because it necessitated the formalization of a note template that facilitated providers’ and scribes’ ability to convey information to one another, to share meaning about what was conveyed, and to integrate the clinical, financial, and legal requirements of the note. Scribing had a generative effect because, in trying to share meaning, the provider and scribe made affordances for each other’s personal and professional differences. Scribing thus generated a mutuality that might not have existed before. That connection allowed the provider to shift her perspective from the computer to the patient, and in so doing, re-engage the patient in the encounter. However, providers and scribes noted the success of a scribing intervention differently. Providers noticed iterative interactions with scribes around concrete activities related to note-taking, as well as revelatory conversations with patients that uncovered more “oh by the ways.” Comparatively, scribes were encouraged by blossoming mutuality with the provider, specifically sharing a sense of being jointly invested in the success of each other as care workers.

These findings support and particularize current research into clinicians’ and patients’ perspectives on scribes in primary care. Yan et al. (2016) describe “interpersonal fit” between scribes and providers as a key feature of successful scribing [16]. Sattler et al. (2018) further note how the interprofessional benefits of scribing on teamwork increase providers’ “joy of practice” [15]. Both researchers also made note of interpersonal benefits for the patient when the providers paid more attention to patients and had a better sense of the patient’s concerns [15, 16]. In describing the collective action of providers and scribes in implementing a scribing practice, we also took note of how scribing allowed providers to re-engage the patient. Based on our findings, we suggest that the patient centeredness that appears to accompany a scribing practice emerges from changes in care practices that emphasize the complementarity of existing professional roles, which thus leverage the interactional possibilities already present in the primary care team. Scribing, as a skill, forged moments of interprofessional fit. Scribing, in practice, created opportunities for interpersonal connection.

### Limitations

When we started the data analysis, our goal was to create individual reports for sites, as well as an amalgamated final report for VHA Patient Care Services, our operational partner. We used a matrix analysis to quickly identify barriers and facilitators to implementation; if our intention had not been to generate meaningful reports, and quickly, our method of initial data analysis might have been different. Normalization Process Theory helped us structure our qualitative findings in a way that illuminated the slow work of implementation, how collective action facilitated incremental changes. We focused on teams that successfully initiated a scribing practice, paying attention to teams that were unsuccessful might provide illuminating counter-examples to those we discuss in this paper. Talking with these other teams might help inform the spread of scribing to a wider array of teams. Additionally, the scope of the article was limited to provider and scribe perspectives in VHA primary care clinics in the United States, which did not allow us to explore the global reach of scribing as a practice, the generalizability to non-VHA primary clinics, or the unique perspectives of Veteran patients. Finally, we were not able to check the content of the note against the patient’s perspective on what information was significant and should have been included in the note; Veterans do have the capability of downloading and reviewing notes in their medical record through MyHealth*e*Vet (the VHA patient portal), but the ability to incorporate that process was outside the scope of our evaluation.

## Conclusions

Moving forward, our research suggests that individuals will notice different benefits to scribing, based on their professional expectations and organizational roles related to documenting patient visits. Providers might notice the value of spending less time on documentation and spending more time face-to-face with their patients. Scribes might notice being trusted and valued as peers. Quantitative measures capable of capturing these characteristics could provide indications of a successful scribing practice in a larger trial. Additional practical recommendations related to the collective action needed to implement a successful scribing practice include:

- Develop a note template for the scribe, which can be provider- or clinic-specific, and may need to address local customs or rules.
- Develop a clear scope of practice for the scribe when working in outpatient clinics.
- Establish a training plan to include using the electronic medical record, medical terminology, and scribing protocol.
- Ensure an “orientation period” to allow the scribe to establish relationships with the provider and the primary care team members.
- Plan for coverage during periods of vacation, sick days, and turnover.

## Additional files

Additional file 1: Semi-Structured Interview Guide used with Providers and Scribes. List of interview questions. (DOCX 14 kb)

Additional file 2: Structured codebook for 6-month site visit data. Blank codebook that lists only codes based on our ethnographic process evaluation aims. (DOCX 17 kb)

## Data Availability

The authors declare that the data supporting the findings of this study are available within the article.

## Abbreviations

CNA: Certified Nursing Assistant
DO: Doctor of Osteopathic Medicine
LPN: Licensed Practical Nurse
MD: Doctor of Medicine
MSA: Medical Support Assistant
NP: Nurse Practitioner
NPT: Normalization Process Theory
PACT: Patient-Aligned Care Team
RN: Registered Nurse
RNCM: Registered Nurse Care Manager
VHA: Veterans Health Administration
