# Supplementary material for: Taking note: A qualitative study of implementing a scribing practice in team-based primary care clinics
Source: BMC Health Serv Res. 2019 Aug 14;19:574. doi: 10.1186/s12913-019-4355-z (PMC6694617; doi:10.1186/s12913-019-4355-z)
Supplement: Supplementary file 1 — Semi-Structured Interview Guide used with Providers and Scribes. List of interview questions. (DOCX 14 kb) [file 12913_2019_4355_MOESM1_ESM.docx]

**Appendix 1:** Semi-Structured Interview Guide used with Providers and Scribes

| **Category** | **Questions** |
| --- | --- |
| Education / Training / Experience Level | 1. Please describe your level of education and/or clinical licensure. 2. How long have you worked at VA? 3. Are you a Veteran? 4. Have you received training in PACT? If so, when? 5. What training on scribing have you and your team received prior to implementing this pilot? Who was trained? When? |
| Team Function | 1. How long has your team been working together? 2. How long has your team been working together with a scribe? 3. Has the pilot affected how you interact with your team? If so, describe. 4. Thinking about the other members of your team, describe ways in which the scribe helps you to do your job and perhaps ways that they could better support you in your role? |
| The Model | 1. Please describe the scribe model at your facility. 2. Please describe how you have implemented scribing on your team.    1. Describe the learning curve. What modifications did you have to make along the way?    2. Did you create a template note for the scribe to use in the EMR?    3. How did you decide what information to capture in the note?    4. How do you translate what happens in the appointment onto the CPRS note template?    5. What tools do you use to scribe (e.g., laptop, dictation device, pen/paper & notebook)? 3. Do you feel that the pilot is having any effect on the quality of an encounter during a typical appointment with a patient? 4. What are the advantages to scribing? 5. What are the main challenges to using a scribe during an appointment? 6. Is the [LPN or MA] the appropriate role for scribe tasks? If so, why? If not, why not? If not, which role would be more appropriate? 7. How do you address the regulatory restrictions around scribing? 8. Have you made changes to the physical space/set up of the exam room to facilitate scribing duties? 9. If another PACT wanted to start a scribing/HA team, what advice would you give them? |
| Clinical Workflow/Productivity | 1. What did you hope to gain or achieve by participating in the pilot? 2. Would you say those goals have been achieved? Please describe. |
| Satisfaction | 1. How would you characterize your job satisfaction pre- and post- implementation? 2. What, if any, impact has the pilot had on how you think about your clinical career? (scribe only) |
