# Supplementary material for: Taking note: A qualitative study of implementing a scribing practice in team-based primary care clinics
Source: BMC Health Serv Res. 2019 Aug 14;19:574. doi: 10.1186/s12913-019-4355-z (PMC6694617; doi:10.1186/s12913-019-4355-z)
Supplement: Supplementary file 2 — Structured codebook for 6-month site visit data. Blank codebook that lists only codes based on our ethnographic process evaluation aims. (DOCX 17 kb) [file 12913_2019_4355_MOESM2_ESM.docx]

**Appendix 2:** Structured codebook for 6-month site visit data

| **Site:** |  |
| --- | --- |
| **Team:** |  |
| **Model:** |  |

| **Descriptions of the model** | |
| --- | --- |
| **By the PCP** |  |
| **By the Scribe** |  |
| **By RNCM** |  |
| **By Other Staff**  **LPN, LPN2, MSA, RN, RN2** |  |
| **Facilitators & Barriers to Implementation** | |
| **Organizational facilitators** |  |
| **Organizational barriers** |  |
| **PACT team facilitators** |  |
| **PACT team barriers** |  |
| **Individual facilitators** |  |
| **Individual barriers** |  |
| **Perceptions of the Model** | |
| **PACT team members** |  |
| **Patients** |  |
| **Impacts of the Model** | |
| **Patient satisfaction** |  |
| **Provider satisfaction** |  |
| **Access** |  |
| **PACT team function** |  |
| **Provider burnout** |  |
| **Clinical workflow and productivity** |  |
| **Quality of patient-provider communication** |  |
| **Additional Contextual Information** | |
| **Unintended consequences** |  |
